# Supplementary material for: Patterns of Intron Gain and Loss in Fungi
Source: PLoS Biol. 2004 Nov 30;2(12):e422. doi: 10.1371/journal.pbio.0020422 (PMC532390; doi:10.1371/journal.pbio.0020422)
Supplement: Table S1 — Also available at http://genes.mit.edu/NielsenEtAl/. (4.3 MB ZIP). [file pbio.0020422.st001.zip › NielsenEtAl/html/214.html]

AN0655.1.NCU06636.1.MG00757.1.FG10100.1


```
 CLUSTAL W (1.82) Multiple Sequence Alignments - Introns Inserted


Sequence 1: AN0655.1	281 aa
Sequence 2: MG00757.1	299 aa
Sequence 3: FG10100.1	303 aa
Sequence 4: NCU06636.1	287 aa
Alignment Length: 308 aa
Number Identitical Residues: 113 aa
Alignment Score (without introns) 5521


MG00757.1 	MDKLLNHAFDNLASYDGAKVRRGLRQVEGLLAPICLAPPVTKDSD------VFNTNSAAN
NCU06636.1	MESLLSLAFDNLSAFEPAKIKKGLKQVEGLLAQICLSGPAKRELD------------AAA
FG10100.1 	MESLLSLAFDNLSSYDGPKVRKGLRQVEGLLAQICLSNANTKAKASPRNDRAHRSRDSDN
AN0655.1  	MEALLAHSFDYLSSYEPTKVRKGLRQVEGLLAQICLS---RAKRP------VSDKRGSPL
          	*: **  :** *:::: .*:::**:******* ***:                 . .:  

MG00757.1 	QAGSPDQPAPRRTLAELAEDPAFRAFFRLQDSFEWNIISRLIDTLN~NLLSRGAEGENDM
NCU06636.1	AGEQPQ--LQRKGLGDLAGDPAFREFFKLQEGFEWNVAQRLLTTLD~FLVARGGDGQYDL
FG10100.1 	DGSDTSSTPPQKDLARLSRDPAFREFFKLQEGFEWNVAMRLISTLD~RLMAKGGDGGNDL
AN0655.1  	LCGAPQ--SGCKALSELRDDPAFREFFKLQDGFQWN---------E1HLLGRENSGTNDL
          	    ..     : *. *  ***** **:**:.*:**         :  *:.:  .*  *:

MG00757.1 	LIVSALELMQGLLLLHPPSKILFMRDPSMN0LLLDLLEPCNCPAIQKAAVLVLVTALIDT
NCU06636.1	LILNALDLIQGVLLLHPPSKVLFSRAAHMN0LLLDLIEPVNTPAIQCATIITLVVALLDS
FG10100.1 	LILSSLDLIQGLLLLHPPSKSLFAREQNMN0LLLDLLEPYNCPAIQSATLLTLVTALIDT
AN0655.1  	LILSTLDLIQGVLLLHPPSRILFAREIYMN~LLLDLLDPINCPAIQSATLLTLVTALLDH
          	**:.:*:*:**:*******: ** *   ** *****::* * **** *:::.**.**:* 

MG00757.1 	PQNTRTFERLDGLLTVTSLFKSRATSRDLKVKLMEFLYFYLMPETASIPRAELRERPSVP
NCU06636.1	PANVRVFERLDGLLTVASLFKSRETGREVKFRLTEFLYFYLTPEAPSASMASSP----VL
FG10100.1 	PINTRTFESLDGLLTITSLFKSRSTAREVKLKLVEFLYFYLMPEIPSIPRADQRD--SVP
AN0655.1  	PANTRTFEELDGLLTVTSLFKQRSTAREVKLKLVEFLYFYLMPETAMVSTSAPN------
          	* *.*.** ******::****.* *.*::*.:* ******* ** .  . :         

MG00757.1 	ALLQRSPSKLAKAFSGDGTPERRSRADS-GSAAILSTEEKQALLARHLS-NVGDLVRDLR
NCU06636.1	PSTPTSPTRDRAGSDTASQQGRGSKANG---GVTLSVDVKKRKLDQHLPGVVDALLRDLD
FG10100.1 	AMFQRSPSKLAGAFNADSRR-KRSGSDP-EGDAYLTTDEKQELLSQHLS-SVEELVKDLR
AN0655.1  	TALQRSPSKLGGGPVSRSVNVSGSHSNGRGSRDTRTTEEKQSLLGRYLH-NVEDLVEDLK
          	.    **::   .    .     * ::   .    :.: *:  * ::*   *  *:.** 

MG00757.1 	SCTPFGVIC-
NCU06636.1	RYKPFGGVLS
FG10100.1 	TCAPFGGVVC
AN0655.1  	ETAPFGATVY
          	   ***
```
